# Congenital Strangulated Transmesenteric Hernia: A Rare Cause of Acute Bowel Obstruction

**Published:** 2013-07-01

**Authors:** Landry Yapi Aké, Nicolas Ello Moh, Abass Kéita, Cosme Aguehoundé

**Affiliations:** University of Cocody (Abidjan, Cote d'Ivoire)

**Keywords:** Intestinal obstruction, Internal hernia, Neonate

## Abstract

A case of congenital transmesenteric hernia leading to intestinal obstruction is being reported here.

## INTRODUCTION

Internal hernia is the protrusion of a viscus, within the peritoneal cavity, through an opening (normal or abnormal) in the peritoneum or the mesentery [1]. The incidence of internal hernias is less than 1% [1, 2] and they represent 5.8% of small bowel obstruction [1, 2]. The hernial opening can be acquired or congenital particularly in the case of transmesenteric and transmesocolic hernias which represent 8% of all internal hernias [3]. Congenital transmesenteric hernias, when strangulated, can cause acute bowel obstruction with potentially fatal consequences.

## CASE REPORT

A 5-week-old female infant, weighing 3.5 kg, born from a full-term twin pregnancy with no prenatal or perinatal risk factors, was admitted in emergency with progressive abdominal distension, bilious vomiting, fever and failure to pass faeces for 3 days. She had pallor and signs of dehydration. Vital signs were normal. The abdomen was distended with generalized tenderness and guarding. Bowel sounds were absent; no mass was palpable. The rectal examination was normal. The plain abdominal X-ray showed features of small bowel obstruction and poor aeration of the distal gut without any sign of pneumoperitoneum. The ultrasound scan showed significant gaseous distension of proximal bowel loops with a minimal intra-abdominal fluid effusion. However, there was no obvious evidence of bowel invagination. A differential diagnosis of midgut volvulus secondary to malrotation and intussusception were considered. Blood results revealed hyponatremia, hyperkaliemia and hypochloridemia with microcytic normochromic anaemia (haemoglobin concentration: 5 Gr/dl). After correction of hydration, electrolyte disorders and blood transfusion, a laparotomy was performed by a supra umbilical transverse approach. A 25 cm long necrosed jejunal loop, starting from 30cm of ligament of Trietz, was found incarcerated in a trans-mesenteric opening of 3 cm diameter; resulting in a strangulated trans-mesenteric internal hernia (Fig. 1). Intestinal resection was performed removing the segment of sphacelus loop along with the mesenteric defect, with an immediate end-to-end anastomosis.

**Figure:**
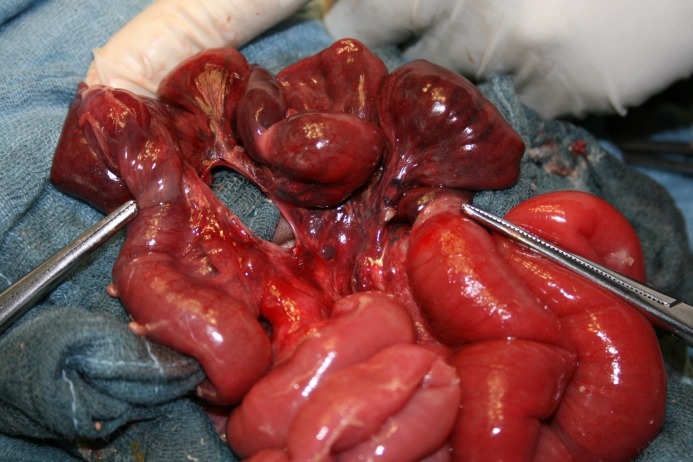
Figure 1: Intraoperative findings after reduction, a round mesenteric defect in the segment of the jejunum with gangrenous loop.

The post-operative period was marked by the occurrence of disseminated intravascular coagulation disorder and dyselectrolytemia, which required management in the intensive care unit. These complications worsened causing death of the infant on the fourth post-operative day.

## DISCUSSION

Internal hernias are rare diseases [1]. They can be acquired following a Roux-en Y- anastomosis which can be post traumatic or post inflammatory [2]. Their origin can be congenital causing transmesenteric hernias in 6.60% of cases, paraduodenal hernias in 2.20% of cases and pericaecal hernias in 2.20% of cases [4]. Congenital transmesenteric hernia is a rare disease which remains the most common aetiology of congenital internal hernias constituting 35% of cases [1, 4].

Patients of any age can be affected by this pathology [5, 6, 7, 8]. The diagnosis is usually made during a laparotomy for acute bowel obstruction due to strangulation [5, 6, 7]. The preoperative diagnosis of a complicated internal hernia is difficult to make, however, in some cases a computed tomography scan can contribute [5, 9]. 

According to Fujita [10], a series of plain abdominal X-Rays can be useful in the diagnosis of this condition by showing a constant image indicating the presence of air in the bowel accompanied by a circular or oval lacuna of the image of gas in the middle of the abdomen.

The size of the mesenteric defect varies from 2 or 3 cm to 15 cm [5]. This defect is either near the ileocaecal region or Treitz’s ligament [1]. According to Capito [6], this defect could be the first step of the formation of a bowel atresia. Its occurrence could be explained by a thrombosis with limited necrosis of the mesentery [6]. Other theories mention the occurrence of intra- peritoneal inflammation, traumatism, partial regression of the development and the induction of a mesenteric opening by the colon during its migration through the umbilical opening [1, 2, 9]. 

There was only one mesenteric defect in our case, but in certain cases there have been two defects as described by Lin [7]. Bowel resection with end-to-end anastomosis and closure of the mesenteric opening should be performed [5,7].

The delay in surgical management led to strangulation and necrosis of bowel in our case. Bowel necrosis is known to contribute to mortality in 40-80% of the cases [5, 7].

## Footnotes

**Source of Support:** Nil

**Conflict of Interest:** None

